# Bacteriophage Therapy for Critical Infections Related to Cardiothoracic Surgery

**DOI:** 10.3390/antibiotics9050232

**Published:** 2020-05-05

**Authors:** Evgenii Rubalskii, Stefan Ruemke, Christina Salmoukas, Erin C. Boyle, Gregor Warnecke, Igor Tudorache, Malakh Shrestha, Jan D. Schmitto, Andreas Martens, Sebastian V. Rojas, Stefan Ziesing, Svetlana Bochkareva, Christian Kuehn, Axel Haverich

**Affiliations:** 1 Department of Cardiothoracic, Transplantation and Vascular Surgery, Hannover Medical School, 30625 Hannover, Germany; Salmoukas.Christina@mh-hannover.de (C.S.); Boyle.Colleen@mh-hannover.de (E.C.B.); Warnecke.Gregor@mh-hannover.de (G.W.); Tudorache.Igor@mh-hannover.de (I.T.); Shrestha.Malakh.lal@mh-hannover.de (M.S.); Schmitto.Jan@mh-hannover.de (J.D.S.); Martens.Andreas@mh-hannover.de (A.M.); Rojas.Sebastian@mh-hanover.de (S.V.R.); Kuehn.Christian@mh-hannover.de (C.K.); Haverich.Axel@mh-hannover.de (A.H.); 2 Lower Saxony Centre for Biomedical Engineering, Implant Research and Development, 30625 Hannover, Germany; 3 Institute for Medical Microbiology and Hospital Epidemiology, Hannover Medical School, 30625 Hannover, Germany; Ziesing.Stefan@mh-hannover.de; 4 G.N. Gabrichevsky Research Institute for Epidemiology and Microbiology, Moscow 125212, Russia; cip1989@gmail.com; 5 German Center for Lung Research (DZL), 30625 Hannover, Germany; 6 Clinical Research Group (KFO 311), German Research Foundation, 30625 Hannover, Germany

**Keywords:** phage therapy, bacterial infection, cardiothoracic surgery, implant-associated infection, transplant-associated infection, surgical site infection

## Abstract

(1) Objective: Bacterial resistance to conventional antibiotic therapy is an increasingly significant worldwide challenge to human health. The objective is to evaluate whether bacteriophage therapy could complement or be a viable alternative to conventional antibiotic therapy in critical cases of bacterial infection related to cardiothoracic surgery. (2) Methods: Since September 2015, eight patients with multi-drug resistant or especially recalcitrant *Staphylococcus aureus*, *Enterococcus faecium*, *Pseudomonas aeruginosa*, *Klebsiella pneumoniae*, and *Escherichia coli* infections were treated with bacteriophage preparations as a therapy of last resort according to Article 37 of the Declaration of Helsinki. Patients had infections associated with immunosuppression after organ transplantation or had infections of vascular grafts, implanted medical devices, and surgical wounds. Individualized phage preparations were administered locally, orally, or via inhalation for different durations depending on the case. All patients remained on conventional antibiotics during bacteriophage treatment. (3) Results: Patients ranged in age from 13 to 66 years old (average 48.5 ± 16.7) with seven males and one female. Eradication of target bacteria was reached in seven of eight patients. No severe adverse side effects were observed. (4) Conclusions: Phage therapy can effectively treat bacterial infections related to cardiothoracic surgery when conventional antibiotic therapy fails.

## 1. Introduction

Patients that have undergone cardiothoracic surgery are at a particularly high risk of life-threatening infectious complications. Surgical site infections substantially contribute to postsurgical morbidity and mortality.

Implant-associated infections often become chronic, as bacteria growing on artificial surfaces tend to form biofilms that are highly tolerant to antibiotics. In addition, drug-induced immunosuppression renders heart and lung transplant patients particularly vulnerable to life-threatening infections. Considering these challenges and the global rise in bacterial resistance to conventional antibiotics, there is a desperate need for new antibacterial agents and strategies.

Bacteriophages (or phages) are viruses that specifically infect bacteria. With the dawn of antibiotics, the notion of using bacteriophages to treat clinical infections was neglected for almost a century except in some Eastern European countries and the former USSR [1,2]. In recent years, revival of the use of lytic phages for hard-to-treat bacterial infections has gained significant interest, however, relatively few phages have shown clinical efficacy. Nevertheless, several recent case studies have reported success using local [3] and parenteral [4] phage therapy with natural bacteriophages, as well as with the genetically engineered bacteriophages [5].

Here, we report a case series of implant- and transplant-associated multi-drug resistant or recalcitrant infections that were successfully treated with individualized bacteriophages. The current case series includes patients who were treated using our recently described strategy of phage application in combination with fibrin glue. Fibrin glue is a two-component hemostat, sealant, and tissue adhesive consisting of fibrinogen and thrombin. In this case, half of the thrombin solution is substituted with phage suspension [6] and the mixture applied intraoperatively to act as a phage-containing biocompatible scaffold or coating. This unique approach allows for the sustained release of phages to infected sites. These results demonstrate that modern phage therapy is a powerful alternative, or viable support, to standard antibiotic therapy for severe infections.

## 2. Results

Details concerning phages and antibiotic administration, as well as microbiological results and survival data are presented in Table 1. The antibiotic regimens for each patient were the same before and during phage therapy. 

Detailed data of inflammation parameters are presented in the Supplementary Tables S1–S8 and Supplementary Figures S1–S8.

### 2.1. Clinical Outcome

Patient 1: After the second phage application, *Staphylococcus aureus*, *Enterococcus faecium*, and *Pseudomonas aeruginosa* were no longer detected and phage therapy was stopped. Bacteria were not detected for 16 days after the last phage application. Unfortunately, the patient developed a subsequent infection caused by *P. aeruginosa* and *E. coli* 17 days after phage therapy, which was treated only with conventional antibiotic therapy one month later in another hospital. It is not known whether the second *P. aeruginosa* isolate was the same as the first *P. aeruginosa* isolate, however, it did have a different antibiogram than the first isolate, which would suggest it was an independent infection.

Patient 2: After phage therapy, *Klebsiella pneumoniae* was not detected in bronchial lavage samples but was found in stool samples. However, in contrast to the pan-resistant strain causing the lung infection, the *K. pneumoniae* strain isolated from the patient’s stool was susceptible to antibiotics. 

**Figure 1:**
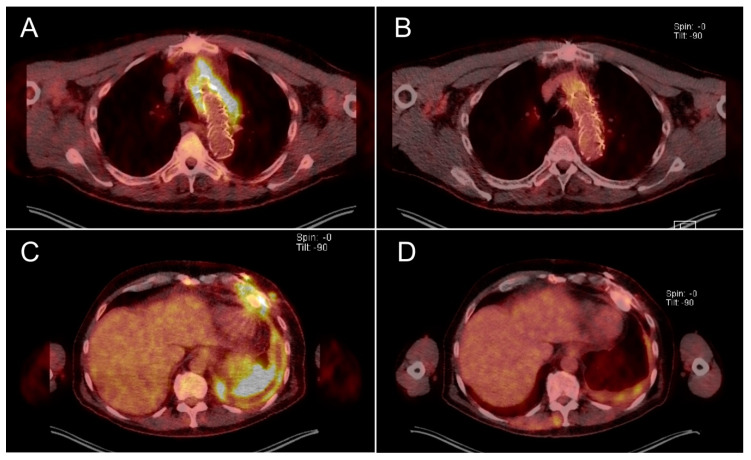
PET-CT scans of Patient 3 before (**A**) and seven months after (**B**) phage therapy in the area of the aortic graft and of Patient 4 before (**C**) and two months after (**D**) phage therapy in the area of the left ventricular assist device (LVAD) and pleural cavity empyema. Yellow emission shows level of accumulation of the tracer substance (2-[^18^F]fluoro-2-desoxy-D-glucose), which corresponds to inflammation.

Patient 3: After the last phage application, blood culture samples were free of *S. aureus*. A positron emission tomography/computed tomography (PET-CT) scan obtained seven months after phage therapy showed no signs of graft infection (Figure 1B).

Patient 4: After phage therapy, no bacteria were detected from wound swabs. The left ventricular assist device (LVAD) remained uninfected which was reflected on a PET-CT scan two months after phage therapy (Figure 1D). Patient 4 showed no further signs of bacterial infection, however, this patient died due to transplant failure 20 months after phage therapy ended. It is extremely unlikely that the transplant failure and subsequent death was related to the previously resolved infection or to phage therapy. 

Patient 5: The in vitro activity of phages was tested throughout phage therapy and there was no evidence of bacterial resistance to the bacteriophage strains used. After the first dose, viable phages were consistently detected in the drainage fluid (≥10^4^ pfu/mL) prior to subsequent phage applications. Up to two weeks after phage application, there were no signs of bacteriophage-neutralizing antibodies in the patient’s serum. Nevertheless, moderate but steady levels of *S. aureus* were detected in the drainage fluid. To potentially improve delivery of the phages to the infection site, surgical intervention was offered but declined by the patient. 

In Patients 6–8, intraoperative application of fibrin glue-bacteriophage preparations onto target devices or tissues resulted in the sustained release bacteriophages.

Patient 6: *S. aureus* was not detected after phage therapy. Observation of the pump 1.5 months after phage application did not show signs of an infection or remnants of the fibrin glue.

Patient 7: The wound completely healed and *E.coli* was no longer detected after phage therapy. 

Patient 8: The wound completely healed and *P. aeruginosa* was not detected after phage therapy.

### 2.2. Safety and Adverse Events

We did not observe any major, minor, or unexpected side effects of phage therapy in our treated patients.

## 3. Discussion

Dr. Victor-Henri Hutinel together with Félix d’Herelle applied bacteriophages for the first time in man in 1919 [7]. In the following decades, clinical use of phages gained popularity and application methods were refined. However, in the early 1940s with the discovery of antibiotics, phage therapy fell into obscurity [8]. Since the beginning of the 21st century, development and spread of multi- or pan-resistant bacteria has become a major health issue, leading to renewed interest in phage therapy. Here we report eight patients who had implant- or transplant-associated multi-drug resistant or recalcitrant infections and were successfully treated with individualized bacteriophages, with complete eradication of the target bacteria in seven of eight patients.

Bacteriophages are known as safe and effective antibacterial agents. They are nontoxic to plants and animals and highly specific in that they do not disrupt the composition of normal microflora [1,2,9]. Modern genetic engineering techniques allow the design of the new therapeutic phages with desired properties, for example, specific destruction of biofilms or lysis of previously incurable pathogens [5,10]. Another advantage of bacteriophages is that they are self-amplifying “auto dosing” drugs since they keep replicating in the presence of susceptible host bacteria.

Recurrent or new bacterial infections after an initial round of phage therapy should not be a reason to avoid repeated courses of phage therapy if suitable phages are available. Patient 1 developed a second infection 17 days after ending phage therapy. At the time, we were not able to perform a rapid selection of phages against the isolated *P. aeruginosa* and *E. coli* due to the absence of a local phage collection. We have recently established a collection of strictly virulent, well-characterized bacteriophages at our clinics which now allows us to respond quickly in such cases. The collection consists of both imported and newly isolated phages. Moreover, nowadays, many research groups and organizations (e.g., DSMZ, Germany) have specialized collections of bacteriophages suitable for clinical application after a proper preparation.

In one case (Patient 2), we observed a relevant change in the antibiotic susceptibility of *K. pneumoniae* isolated later from stool (Supplementary Figure S9). The success of phage therapy in some cases can be explained by resensitizing bacteria to antibiotics under phage therapy-induced evolutionary pressure [11].

One major challenge of phage therapy is the delivery of phages to the desired site. The inefficiency of phage therapy of Patient 5 can be explained by complications of delivery of bacteriophages to all infected sites via a drainage and the presence of a preformed biofilm on the surface of the LVAD. The failure to completely eradicate the infection in this case made us consider alternative ways to deliver phages to patients at high risk of reinfection in the early postoperative period. We recently developed and tested medical fibrin sealant as a local and sustained phage delivery system in vitro [6]. We now report three successful first-in-man applications of fibrin glue-embedded bacteriophage preparations for sustained delivery of bacteriophages to infection sites and protection of the implant surfaces, as well as the surrounding tissue from reinfection.

Mammals elicit a humoral immune response to bacteriophages. Development of anti-bacteriophage antibodies can prevent the long-term efficacy phage therapy [12]. We performed the phage neutralization test [12] using serum from Patient 5 in order to assess this issue of phage therapy but did not find any neutralizing activity.

Patients 1, 2, 3, 4, 6, and 7 had elevated CRP levels shortly after phage therapy which decreased within the next few days, similar to the previous experiences [13,14]. This can be explained either by normal postoperative conditions or by significant bacterial lysis due to phage therapy. On the one hand, an increase in inflammation can be considered as a possible downside of phage therapy. However, on the other hand, such a reaction can also be necessary to clear the infection and has also been observed with antibiotic therapy. Moreover, modern dialysis and blood filtration systems can often efficiently treat septic patients. Therefore, this effect should not be a reason to not apply phage therapy.

Our clinical results support the growing data that individualized phage therapy is a promising therapeutic approach for patients suffering from bacterial infections that do not respond to conventional antibiotic therapy. However, measures such as surgical debridement, local drug delivery systems, and repeated courses of phage application are vital for clinical success in cases of surgical infections related to implanted medical devices or transplants.

## 4. Materials and Methods 

### 4.1. Phage Preparation

Potentially suitable bacteriophage strains were selected from the well-characterized collection housed in the Gabrichevsky Institute (Table 2). Lysis efficacy was evaluated by serial dilution spot testing and efficiency of plating was analyzed by the double layer plaque assay [15,16]. Phages with strong lytic capacity and ability to propagate on bacteria isolated from the patients were chosen for therapy. All therapeutic bacteriophage preparations were produced according to a previously established protocol with slight modifications [6,17]. Briefly, a fresh overnight broth culture of relevant host bacteria was inoculated on top of a solid nutrient media free of animal-derived material inside Roux flasks. LB Broth Vegitone (Sigma-Aldrich, USA) was utilized with w/v 2% of agar-agar (Carl Roth GmbH, Germany). After a 2.5–3.5 h incubation at 37 °C, excess liquid was discarded from the flasks, the phages were inoculated in separate Roux flasks on top of the preformed growing lawn, and the flasks were incubated for 12–15 h at 37 °C. Amplified bacteriophages were washed from the agar surface with 5–10 mL of equilibration buffer (Hyglos GmbH, Germany; BioVendor GmbH, Germany) and bacteria were removed by filtration through a 0.22 µm polyethersulfone syringe filter (Sarstedt AG, Germany). Cell-free phage lysates were concentrated and purified using Vivaspin 20 ultrafiltration units with a molecular weight cutoff of 100 kDa for *Podoviridae* and *Siphoviridae* or 1000 kDa for *Myoviridae* bacteriophages (Sartorius AG, Germany). After the final concentration step, purified bacteriophages were resuspended in sterile medical-grade 0.9% NaCl. Phage lysates of Gram-negative bacteria were additionally purified with the EndoTrap HD (Hyglos GmbH, Germany; BioVendor GmbH, Germany) affinity columns before ultrafiltration. The production procedures and characterization of phage strains were performed taking into account the modern principles of quality and safety for phage therapy products [18].

The preparation of a mixture of fibrin glue and phages was performed within the operation room directly before the application with the previously described protocol [6]. Briefly, a two-component fibrin sealant (Tisseel, Baxter, USA) was defrosted and half of the thrombin solution volume was substituted with a relevant phage suspension followed by gentle inverting to mix. The phage application was performed using the Tisseel Spray Set with an air pressure 1.5 bar.

### 4.2. Pre-Study Evaluation

Patients with various bacterial infections of different etiology underwent individualized phage therapy according to Article 37 of the Declaration of Helsinki using the following criteria:

- pan-resistance of the bacterial agent to all available antibiotics;
- complication of the clinical picture despite continuous therapy with antibiotics deemed appropriate by an antibiogram; or
- repetitive medical device infection despite appropriate antibiotic and surgical therapy.

The patients ranged from 13–66 years old (average 48.5 ± 16.7) with 7 males and 1 female. Informed consent was obtained from all treated patients. All relevant information concerning diagnoses, localization of infection, isolated bacterial species, and administration of phage therapy is presented in Table 1. Patients were numbered in chronological order. In the Methods section below, we have cases according to the infection type.

### 4.3. Patients with Infected Vascular Grafts

Patient 1 was in critical condition due to an infected aortic arch prosthesis 2 years after previous Stanford type-A aortic dissection. In the following 2 years, the patient was admitted to our clinic twice due to repetitive graft infection. As a consequence, the patient developed pleural empyema followed by a purulent infection of a bronchial tree via a fistula caused by *S. aureus*, *E. faecium,* and *P. aeruginosa*. The infection was unresponsive to conventional antibiotic therapy. The patient showed a normal body temperature, a leukocyte count of 7.0 × 10^9^/L, a serum C-reactive protein (sCRP) value of 86.2 mg/L, and a serum procalcitonin (sPCT) value of 0.2 µg/L. A phage cocktail was administrated once via a pigtail drainage positioned close to the aortic arch and, on the same day, once orally. Two days later, the patient underwent a thoracotomy and decortication of the pleural empyema and a phage cocktail was locally applied intraoperatively.

Patient 3 suffered from an infected aortic graft after Stanford type-A dissection confirmed by *S. aureus*-positive blood culture and PET-CT scan (Figure 1A). Infection was unresponsive to conventional antibiotic therapy. Body temperature measured 36.6 °C, leukocyte count was 4.7 × 10^9^/L, sCRP was 31.6 mg/L, and sPCT was 0.2 µg/L. The patient received phages via a chest tube inserted under CT control close to the infected graft.

### 4.4. Patients with Infected, Implanted, Metallic Medical Devices

Patient 4 presented with a fulminant left-sided pleural empyema caused by *S. aureus* after implantation of a left-ventricular assist device (LVAD). Figure 1C illustrates the degree of inflammation in the infected area despite antibiotic therapy. Body temperature measured 36.5 °C, leukocyte count was 15.3 × 10^9^/L, sCRP was 62.4 mg/L, and sPCT was 0.1 µg/L. Bacteriophages were applied twice per day for 1 week via a chest tube inserted during an operation for decortication of the empyema.

Patient 5 experienced a chronic LVAD infection by *S. aureus* 4 months after device implantation. The patient had a case history of chronic *S. aureus* carriage in the nose and throat and suffered *S. aureus* septicemia several years beforehand. Before phage application, the patient had a normal body temperature, a leukocyte count of 8.3 × 10^9^/L, sCRP of 29.8 mg/L, and sPCT of 1.2 µg/L. A phage cocktail was applied locally, intranasally, and orally for a prolonged period.

Patient 6 had *S. aureus* infections of a treprostinil pump required for permanent therapy of pulmonary hypertension. The repetitive infection occurred despite reimplantation, surgical debridement, and conventional antibiotic therapy. Before phage therapy, body temperature was normal, leukocyte count was 6.1 × 10^9^/L, sCRP was 6.0 mg/L, and sPCT was <0.1 µg/L. Fibrin glue-embedded bacteriophages were used to cover a new pump which was implanted after surgical debridement of the infected area. To do this, half of the thrombin solution was substituted by a high titer phage solution. The phage-thrombin mixture was combined with fibrinogen in a standard syringe attached to a spray applicator. In total, 4 mL of fibrin glue was used to cover the treprostinil pump surface.

### 4.5. Patients Infected During Drug-Induced Immunosuppression After Organ Transplantation

Patient 2 had a *K. pneumoniae* lung infection after heart transplantation. The patient’s immunosuppression regime consisted of mycophenolic acid, tacrolimus, and prednisolone. The antibiogram of the *Klebsiella* species isolated from the lungs and intestine were both pan-resistant against all relevant antibiotics. The patient had a normal body temperature, leukocyte count of 21.2 × 10^9^/L, sCRP of 49.0 mg/L, and sPCT of 6.3 µg/L. Phages were applied once per day via inhalation and via a nasogastric tube for two days. Subsequently, the number of applications increased to two times per day for two more days.

Patient 8 had a *P. aeruginosa* infected thoracotomy wound two months after a double lung transplantation for cystic fibrosis. For immunosuppression, the patient received mycophenolic acid, tacrolimus, and prednisolone. The patient had a normal body temperature, a leukocyte count of 4.8 × 10^9^/L, sCRP of 113 mg/L, and sPCT < 0.1 µg/L. *P. aeruginosa* was isolated from the wound surface and was not eradicated despite surgical debridement, vacuum-assisted therapy, and continuous antibiotic therapy. The patient received 4 mL of phage-containing fibrin glue sprayed over the wound surface during debridement.

### 4.6. Patient with a Deep Wound Infection

Patient 7 had a deep sternal wound infection caused by *Escherichia coli* after coronary artery bypass surgery and mitral valve replacement. Surgical debridement, vacuum-assisted closure therapy, and over 2 weeks of antibiotic therapy were not successful in eradicating bacteria from the wound. The patient did not have a fever but had a leukocyte count of 6.9 × 10^9^/L and a sCRP of 40.9 mg/L. Phage-embedded fibrin glue (4 mL) was sprayed over the wound once, intraoperatively.

## Figures and Tables

**Table 1:** Summary of phage therapy application and outcomes.

Patient Data, Infection Site, and Date of Surgery ^1^	Source and Date of Isolated Bacteria	Basis for Phage Therapy	Titer and Bacteriophage(s)	Date, Dosage, and Route of Phage Administration	Antibiotic Therapy Before & During Phage Application	Microbiological Control After Phage Therapy	Survival After Phage Therapy
Patient 1, 52 y.o., m. Prosthetic infection after aortic arch replacement. Replacement: 03.12.2013.	Implant drainage Since 20.08.2015: *S. aureus*, *E. faecium.* Since 09.09.2015: *P. aeruginosa.* Bronchial lavage Since 27.08.2015: *E. faecium*, *P. aeruginosa.*	Continuous isolation of *S. aureus*, *E. faecium*, *P. aeruginosa* despite conventional antibiotic therapy	1 × 10^8^ pfu/mL *Staphylococcus* phage CH1 *Enterococcus* phage Enf1 *Pseudomonas* phage PA5 *Pseudomonas* phage PA10	10.09.2015: One 25 mL local application with 6 mL gentamicin (240 mg) and 20 mL daptomycin (350 mg) via drainage One 50 mL per os 12.09.2015: 25 mL locally, intraoperatively	2000 mg cefepime, 500 mg daptomycin, 600 mg linezolid, tobramycin depending on drug concentration in blood (target concentration 2 mg/L). All antibiotics intravenously once per day.	*S. aureus*, *E. faecium*, and *P. aeruginosa* not detected	Died 2 months after phage therapy due to a new bacterial infection caused by *E. coli* and *P. aeruginosa*
Patient 2, 40 y.o., m. Lung infection during drug-induced immuno-suppression after heart transplantation. Transplantation: 23.07.2016.	Bronchial lavage Since 16.08.2016: pan-resistant *K. pneumoniae*. Rectal swab Since 27.06.2016: pan-resistant *K. pneumoniae.*	Infection with the pan-resistant bacteria	1 × 10^8^ pfu/mL *Klebsiella* phage KPV811 *Klebsiella* phage KPV15	29.08.2016–30.08.2016: 2 mL inhalation once per day (mornings) 18 mL via nasogastric tube once per day (mornings) 31.08.2016–01.09.2016: 2 mL inhalation two times per day (mornings and evenings) 18 mL via nasogastric tube two times per day (mornings and evenings)	2000 mg ceftazidime, 600 mg linezolid, 500 mg avibactam intravenously twice per day. Inhalation of 1 MIU colistin three times per day. 2000 mg meropenem intravenously three times per day. 960 mg cotrimoxazole per os once per day. Tobramycin depending on drug concentration in blood (target concentration 2 mg/L).	*K. pneumoniae* not detected in bronchial lavage	Until present
Patient 3, 59 y.o., m. Chronic vascular graft infection after aortic arch replacement. Replacement: 22.10.2014.	Blood culture Since 19.12.2016: *S. aureus*	Continuous isolation of *S. aureus* and high inflammation parameters despite conventional antibiotic therapy	1 × 10^9^ pfu/mL *Staphylococcus* phage CH1	06.01.2017–08.01.2017: 20 mL local application via drainage every 12 hours (4 doses)	600 mg rifampicin intravenously twice per day. 2000 mg flucloxacillin intravenously four times per day.	*S. aureus* not detected	Until present
Patient 4, 62 y.o., m. Fulminant pleural empyema after LVAD implantation. Implantation: 21.04.2017.	Wound swab Since 19.06.2017: *S. aureus*	Continuous isolation of *S. aureus* and high inflammation parameters despite conventional antibiotic therapy	1 × 10^9^ pfu/mL *Staphylococcus* phage CH1	30.06.2017–06.07.2017: 20 mL local application via drainage every 12 hours (14 doses)	500 mg daptomycin intravenously once per day.	*S. aureus* not detected	Died 20 months after heart transplantation due transplant failure
Patient 5, 51 y.o., m. Chronic LVAD infection. Implantation: 28.03.2017.	Implant drainage Since 25.07.2017: *S. aureus* Nasal swab Since 28.05.2014: *S. aureus* Throat swab Since 27.01.2015: *S. aureus*	Continuous isolation of *S. aureus* and high inflammation parameters despite conventional antibiotic therapy	1 × 10^9^ pfu/mL *Staphylococcus* phage Sa30 *Staphylococcus* phage CH1 *Staphylococcus* phage SCH1 *Staphylococcus* phage SCH111	09.08.2017–17.08.2017: 10 mL local application via drainage once per day after flushing with antiseptics and antibiotics 2 mL intranasal once per day and 10–20 mL per os once per day 18.08.2017–23.08.2017: 10 mL local application via drainage every 12 hours after flushing with antiseptics and antibiotics 10–20 mL per os once per day	500 mg daptomycin intravenously once per day.	100× reduction of *S. aureus* in the drainage fluid. Complete eradication of *S. aureus* from nose and throat	Died 1.5 months after beginning phage therapy due to *S. aureus* sepsis
Patient 6, 45 y.o., m. Repetitive treprostinil pump infection. First implantation: 08.08.2017. Second implantation: 12.09.2017.	Catheter Since 16.11.2017: *S. aureus* Blood culture Since 16.11.2017: *S. aureus*	Continuous isolation of *S. aureus* and pump reinfection despite conventional antibiotic and surgical therapy	4 × 10^10^ pfu/mL *Staphylococcus* phage Sa30	29.11.2017: 4 mL locally, intraoperatively mixed with fibrin glue (Tisseel, Baxter, USA)	375 mg sultamicillin two times per day per os.	Not tested	Until present
Patient 7, 66 y.o., f. Sternal wall healing disorder after mitral valve replacement and aortocoronary bypass surgery. Surgery: 23.03.2018.	Wound swab Since 20.04.2018: *E. coli*	Continuous isolation of *E. coli* and high inflammation parameters despite conventional antibiotic therapy	4 × 10^10^ pfu/mL *Escherichia* phage ECD7 *Escherichia* phage V18	09.05.2018: 4 mL locally, intraoperatively mixed with fibrin glue (Tisseel, Baxter, USA)	600 mg clindamycin three times per day per os.	*E. coli* not detected	Until present
Patient 8, 13 y.o., m. Sternal wound abscesses after double lung transplantation. Transplantation: 10.03.2018.	Wound swab Since 27.05.2018: *P. aeruginosa*	Continuous isolation of *P. aeruginosa* and high inflammation parameters despite conventional antibiotic therapy	4 × 10^10^ pfu/mL *Pseudomonas* phage PA5 *Pseudomonas* phage PA10	13.06.2018: 4 mL locally, intraoperatively mixed with fibrin glue (Tisseel, Baxter, USA)	2 MIU colistin intravenously twice per day. 750 mg ceftazidime, 187.5 mg avibactam intravenously three times per day.	*P. aeruginosa* not detected	Until present

^1^ f., female; m., male; and y.o., years old

**Table 2:** List of bacteriophage strains.

Bacteriophage Name	Taxonomy	GenBank Accession Number	Isolation Source	Source
*Enterococcus phage* Enf1	Order *Caudovirales*; family *Siphoviridae*; genus *Sap6virus*	MK800154.1	Wastewater, Moscow, Russia	This study
*Escherichia phage* ECD7	Order *Caudovirales*; family *Myoviridae*; subfamily *Tevenvirinae*; genus *Rb49virus*	KY683735.1	Chicken feces, Moscow Region, Russia	[17,19]
*Escherichia phage* V18	Order *Caudovirales*; family *Myoviridae*; subfamily *Vequintavirinae*; genus *V5virus*	KY683736.1	Cowshed sewage, Moscow Region, Russia	[17,19]
*Pseudomonas phage* PA5	Order *Caudovirales*; family *Myoviridae*; genus *Pbunavirus*	KY000082.1	Wastewater, Moscow region, Russia	[6,20]
*Pseudomonas phage* PA10	Order Caudovirales; family *Myoviridae*; genus *Pakpunavirus*	KY000083.1	Wastewater, Moscow region, Russia	[20]
*Staphylococcus phage* Sa30	Order *Caudovirales*; family *Myoviridae*; subfamily *Spounavirinae*; genus *Kayvirus*	MK331931.1	Clinical material, Astrakhan, Russia	This study
*Staphylococcus phage* CH1	Order *Caudovirales*; family *Myoviridae*; subfamily *Spounavirinae*; genus *Kayvirus*	MK331930.1	Patient’s wound, Chelyabinsk, Russia	[17,19]
*Staphylococcus phage* SCH1	Order *Caudovirales*; family *Podoviridae*; subfamily *Picovirinae*; genus *P68virus*	KY000084.1	Clinical material, Chelyabinsk, Russia	[20]
*Staphylococcus phage* SCH111	Order *Caudovirales*; family *Podoviridae*; subfamily *Picovirinae*; genus *P68virus*	KY000085.1	Clinical material, Moscow, Russia	[20]
*Klebsiella phage* KPV811	Order *Caudovirales*; family *Podoviridae*; subfamily *Autographivirinae*; genus *Drulisvirus*	KY000081.1	Wastewater, Moscow region, Russia	[20]
*Klebsiella phage* KPV15	Order *Caudovirales*; family *Myoviridae*; subfamily *Tevenvirinae*; genus *Jiaodavirus*	KY000080.1	Wastewater, Moscow region, Russia	[20]

## Supplementary Materials

The following are available online at https://www.mdpi.com/2079-6382/9/5/232/s1, Table S1: Patient 1, Figure S1: Patient 1, Table S2: Patient 2, Figure S2: Patient 2, Table S3: Patient 3, Figure S3: Patient 3, Table S4: Patient 4, Figure S4: Patient 4, Table S5: Patient 5, Figure S5: Patient 5, Table S6: Patient 6, Figure S6: Patient 6, Table S7: Patient 7, Figure S7: Patient 7, Table S8: Patient 8, Figure S8: Patient 8, Figure S9: Antibiogram from Patient 2.

## References

[1] Sulakvelidze A., Alavidze Z., Morris J.G. (2001). Bacteriophage therapy. Antimicrob. Agents Chemother..

[2] Summers W.C. (2001). Bacteriophage therapy. Annu. Rev. Microbiol..

[3] Fish R., Kutter E., Wheat G., Blasdel B., Kutateladze M., Kuhl S. (2016). Bacteriophage treatment of intransigent diabetic toe ulcers: A case series. J. Wound Care.

[4] Schooley R.T., Biswas B., Gill J.J., Hernandez-Morales A., Lancaster J., Lessor L., Barr J.J., Reed S.L., Rohwer F., Benler S. (2017). Development and use of personalized bacteriophage-based therapeutic cocktails to treat a patient with a disseminated resistant Acinetobacter baumannii infection. Antimicrob Agents Chemother..

[5] Dedrick R.M., Guerrero-Bustamante C.A., Garlena R.A., Russell D.A., Ford K., Harris K., Gilmour K.C., Soothill J., Jacobs-Sera D., Schooley R.T. (2019). Engineered bacteriophages for treatment of a patient with a disseminated drug-resistant Mycobacterium abscessus. Nat. Med..

[6] Rubalskii E., Ruemke S., Salmoukas C., Aleshkin A., Bochkareva S., Modin E., Mashaqi B., Boyle E.C., Boethig D., Rubalsky M. (2019). Fibrin glue as a local drug-delivery system for bacteriophage PA5. Sci. Rep..

[7] Summers W.C. (1999). Félix d’Herelle and the Origins of Molecular Biology.

[8] Merril C.R., Scholl D., Adhya S.L. (2003). The prospect for bacteriophage therapy in Western medicine. Nat. Rev. Drug Discov..

[9] Kutter E., De Vos D., Gvasalia G., Alavidze Z., Gogokhia L., Kuhl S., Abedon S.T. (2010). Phage therapy in clinical practice: Treatment of human infections. Curr. Pharm. Biotechnol..

[10] Lu T.K., Collins J.J. (2007). Dispersing biofilms with engineered enzymatic bacteriophage. Proc. Natl. Acad. Sci. USA.

[11] Chan B.K., Sistrom M., Wertz J.E., Kortright K.E., Narayan D., Turner P.E. (2016). Phage selection restores antibiotic sensitivity in MDR Pseudomonas aeruginosa. Sci. Rep..

[12] Łusiak-Szelachowska M., Zaczek M., Weber-Dąbrowska B., Międzybrodzki R., Kłak M., Fortuna W., Letkiewicz S., Rogóż P., Szufnarowski K., Jończyk-Matysiak E. (2014). Phage neutralization by sera of patients receiving phage therapy. Viral Immunol..

[13] Jończyk-Matysiak E., Łusiak-Szelachowska M., Kłak M., Bubak B., Międzybrodzki R., Weber-Dąbrowska B., Żaczek M., Fortuna W., Rogóż P., Letkiewicz S. (2015). The effect of bacteriophage preparations on intracellular killing of bacteria by phagocytes. J. Immunol. Res..

[14] Samokhin A.G., Fedorov E.A., Kozlova Y.N., Tikunova N.V., Pavlov V.V., Morozova V.V., Kretien S.O. (2016). Application of the lytic bacteriophages during surgical treatment of the periprosthetic infection of the hip joint endoprosthesis (pilot study). Sovrem. Probl. Nauk. Obraz..

[15] Hyman P., Abedon S.T. (2010). Bacteriophage host range and bacterial resistance. Adv. Appl. Microbiol..

[16] Kutter E. (2009). Phage host range and efficiency of plating. Methods Mol. Biol..

[17] Aleshkin A.V., Volozhantsev N.V., Svetoch E.A., Kiseleva I.A., Rubal’sky E.O., Afanas’ev S.S., Borzilov A.I., Zatevalov A.M., Vasil’ev D.A., Zolotukhin S.N. (2016). Bacteriophages as probiotics: Phage-based probiotic dietary supplement in prophylaxis against foodborne infections. Infect. Dis. Infekt. Bolezn..

[18] Pirnay J.P., Blasdel B.G., Bretaudeau L., Buckling A., Chanishvili N., Clark J.R., Corte-Real S., Debarbieux L., Dublanchet A., De Vos D. (2015). Quality and safety requirements for sustainable phage therapy products. Pharm. Res..

[19] Aleshkin A.V., Rubalskii E.O., Volozhantsev N.V., Verevkin V.V., Svetoch E.A., Kiseleva I.A., Bochkareva S.S., Borisova O.Y., Popova A.V., Bogun A.G. (2015). A small-scale experiment of using phage-based probiotic dietary supplement for prevention of E. coli traveler’s diarrhea. Bacteriophage.

[20] Aleshkin A.V., Ershova O.N., Volozhantsev N.V., Svetoch E.A., Popova A.V., Rubalskii E.O., Borzilov A.I., Aleshkin V.A., Afanas’ev S.S., Karaulov A.V. (2016). Phagebiotics in treatment and prophylaxis of healthcare-associated infections. Bacteriophage.

