# Supplementary material for: Bacteriophage Therapy for Critical Infections Related to Cardiothoracic Surgery
Source: Antibiotics (Basel). 2020 May 5;9(5):232. doi: 10.3390/antibiotics9050232 (PMC7277081; doi:10.3390/antibiotics9050232)
Supplement: Supplementary file 1 [file antibiotics-09-00232-s001.pdf]

Supplementary materials

Bacteriophage therapy for critical infections related to cardiothoracic surgery

Evgenii Rubalskii[1,2*], Stefan Ruemke[1,2*], Christina Salmoukas[1,2], Erin C. Boyle[1], Gregor Warnecke[1], Igor Tudorache[1], Malakh Shrestha[1], Jan Schmitto[1], Andreas Martens[1], Sebastian V. Rojas[1], Stefan Ziesing[3], Svetlana Bochkareva[4], Christian Kuehn[1,2,5,6#], Axel Haverich[1,2,5,6#]

Supplementary Table S1 - Patient 1

Date	sCRP. (mg/L)	Leukocytes. ($\times 10^9$/L)	Body temperature. (°C)	sPCT. (µg/L)
20.08.2015	79	9.00		
21.08.2015	80	7.40		
22.08.2015	74	7.00		
23.08.2015	67	6.70		
24.08.2015	55	6.20		
25.08.2015	52	5.30		
26.08.2015	45	5.50		
27.08.2015	42	5.50	37	0.1
28.08.2015	54	6.30		
29.08.2015	53	5.10		
30.08.2015	54	6.10		
31.08.2015	86	4.90		
01.09.2015	99	5.20		
02.09.2015	122	4.50		
03.09.2015	148	5.70		0.4
04.09.2015	169	4.00		
05.09.2015	154	4.90		
06.09.2015	80	6.80		
07.09.2015	81	6.30		
08.09.2015	94	6.50		
09.09.2015	88	6.70		
10.09.2015	86.2	7.00	37.3	0.2
11.09.2015	120	12.40	37.4	1.6
12.09.2015*	228	14.20	37.6	1.9
13.09.2015	258	9.80	37.9	1.6
14.09.2015	250	9.00	37.6	1.2
15.09.2015	218	7.20	36.6	0.4
16.09.2015	102	6.00	37.2	0.3
17.09.2015	61	8.10	36.7	0.2
18.09.2015	63	4.90	36.3	0.1

19.09.2015	41	8.80	37.2	0.1
20.09.2015	32	5.80	36.2	0.1
21.09.2015	36	9.10	37	0.1

Red color indicates days of phage therapy

*Left-sided re-thoracotomy with suturing of a broncho-pleural fistula

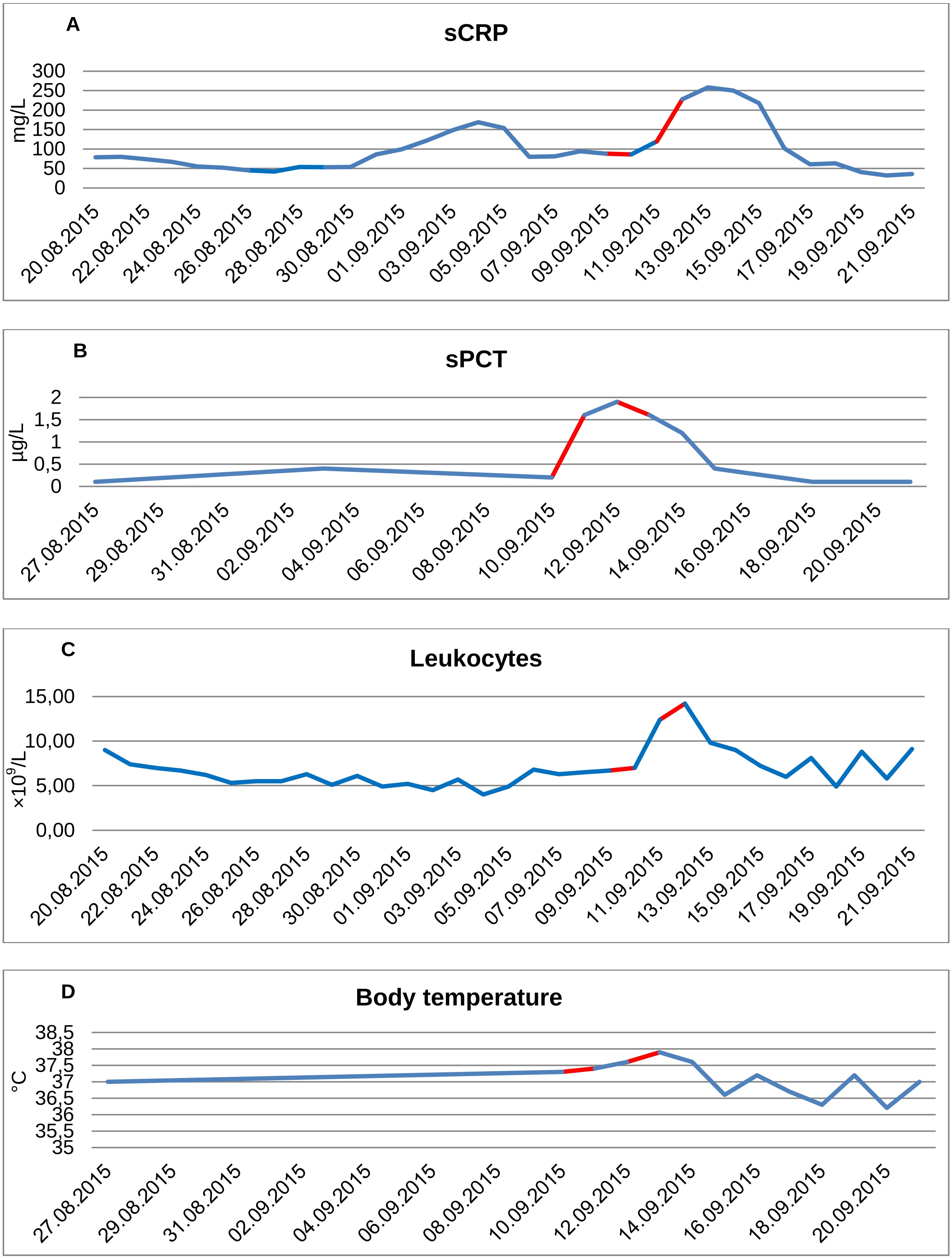

Supplementary Figure S1 – Patient 1

Supplementary Table S2 - Patient 2

Date	sCRP (mg/L)	Leukocytes ($\times 10^9$/L)	Body temperature (°C)	sPCT (µg/L)
23.08.2016	4	34.90	36	2.1
24.08.2016	5	24.50	36	2.2
25.08.2016	7	23.90	36	3
26.08.2016	17	24.20	36.5	3.3
27.08.2016	9	22.80	36	3
28.08.2016	21	22.80	36	2.7
29.08.2016	49	21.20	36	6.3
30.08.2016	83	16.2	36.6	5.8
31.08.2016	116	15	36	6.3
01.09.2016	76	13.7	36.5	4
02.09.2016	55	11.3	35.8	2.8
03.09.2016	42	8.5	35.5	2
04.09.2016	24	10	36.5	2
05.09.2016	20	9.9	37	1.9
06.09.2016	18	12.5	36.1	af
07.09.2016	21	16.3	37.2	2.1
08.09.2016	32	13.4	37	2.3

Red color shows days of phage therapy

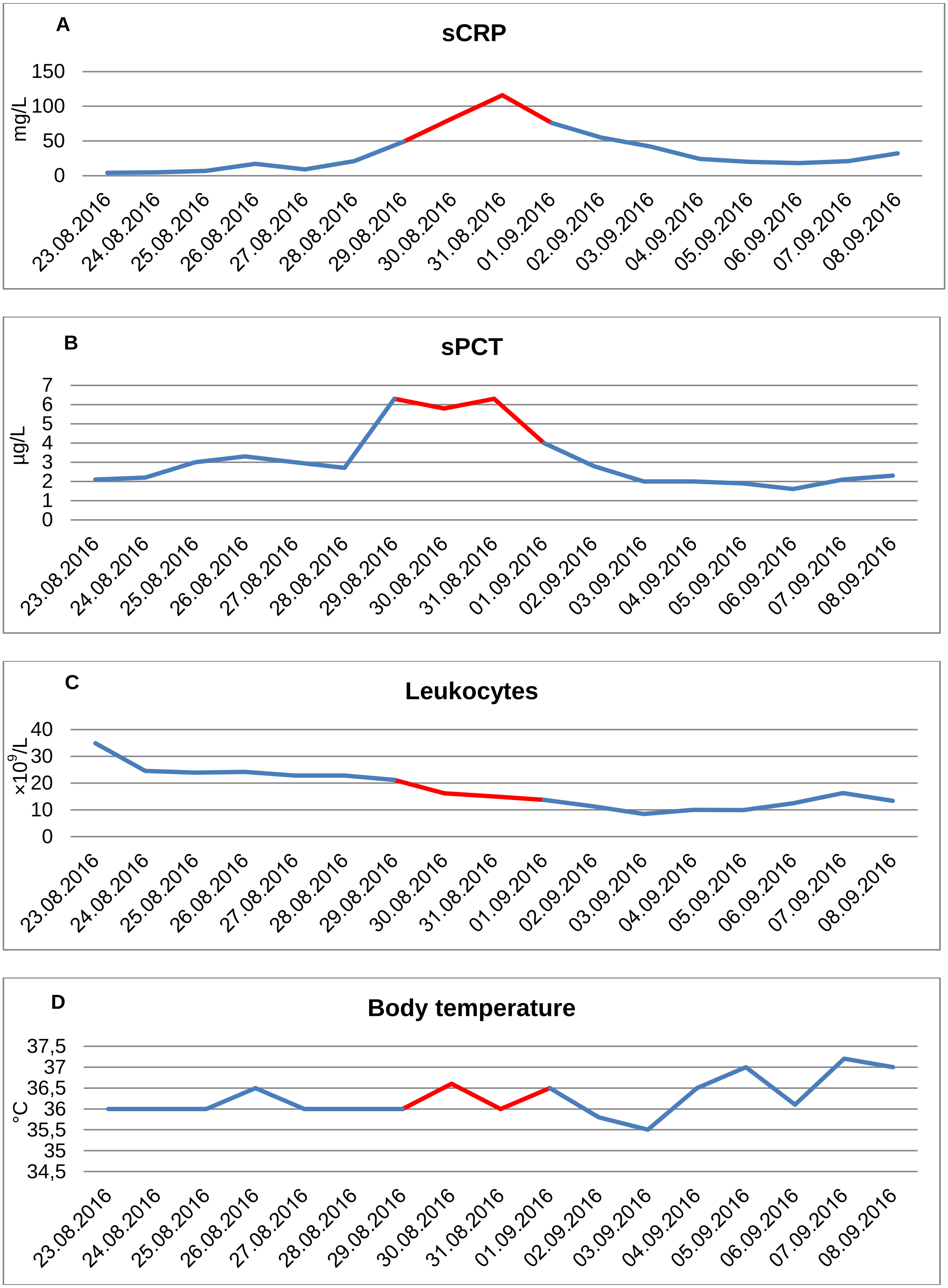

Supplementary Figure S2 – Patient 2

Supplementary Table S3 - Patient 3

Date	sCRP (mg/L)	Leukocytes ($\times 10^9$/L)	Body temperature (°C)	sPCT (µg/L)
19.12.2016				1.1
23.12.2016	183.8	6.00	36.6	
24.12.2016	96.2	6.30	36.7	
25.12.2016	72.9		37	
26.12.2016	69.4		36.6	
30.12.2016	47.3	5.50	36.7	
31.12.2016	50.6	5.20	36.2	0.2
01.01.2017	32.8	5.00	36.8	
02.01.2017	42.5	5.3	36.6	
03.01.2017	40.9	5.1	36.6	
04.01.2017	33.6	4.7	36.5	
05.01.2017	31.6	4.7	36.6	
06.01.2017	26.6	4.2	36.6	
08.01.2017	67.7	6.2	37.8	
09.01.2017	141.9	5.5	37.2	
10.01.2017	137.9	4.6	37	
12.01.2017	39.2	4.5	36.5	0.1
13.01.2017	35.3		36.2	
16.01.2017	22.1	7.7	36.7	
18.01.2017	18	6.3	36.9	

Red color shows days of phage therapy

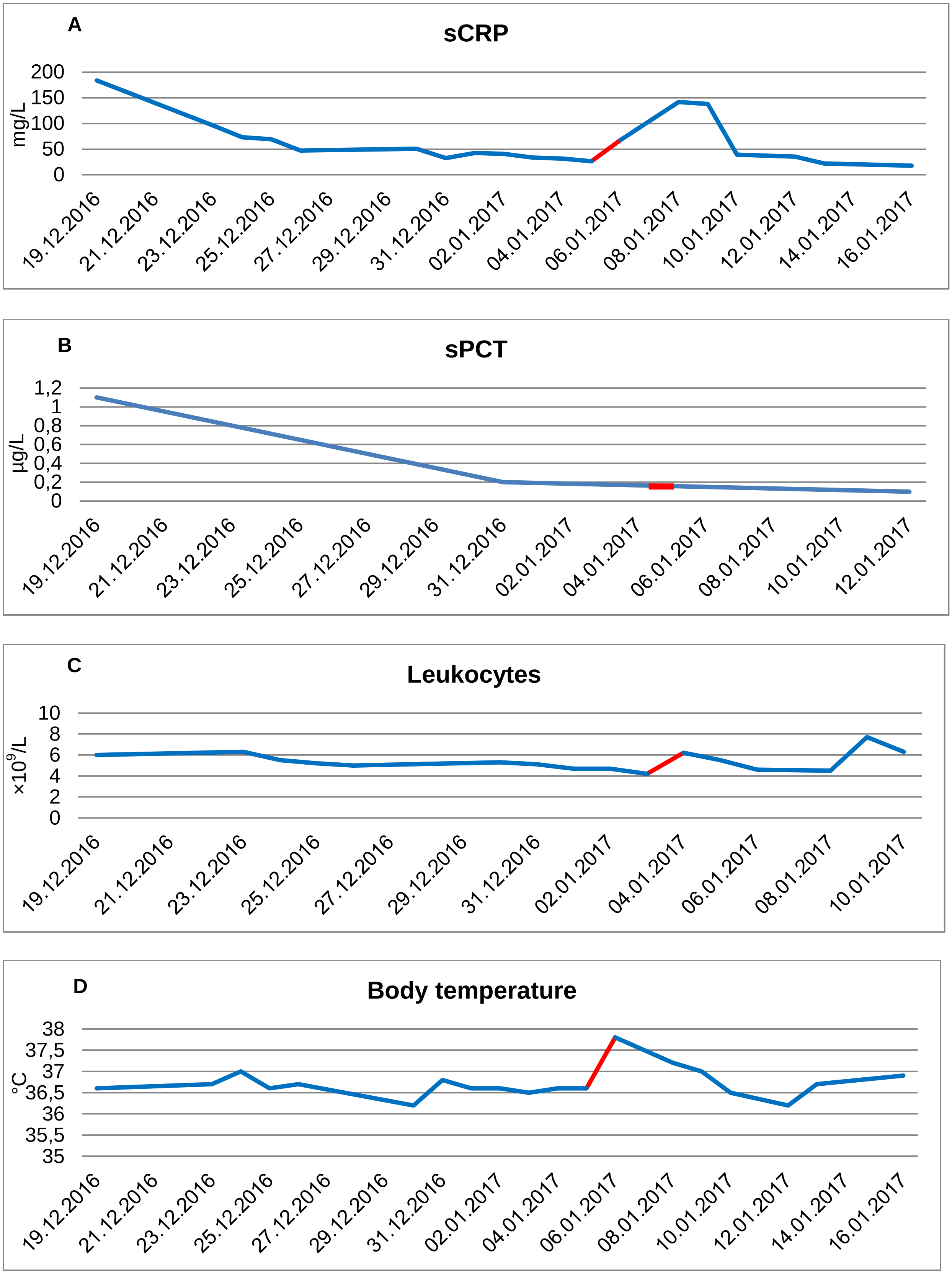

Supplementary Figure S3 – Patient 3

Supplementary Table S4 - Patient 4

Date	sCRP (mg/L)	Leukocytes (×10⁹/L)	Body temperature (°C)	sPCT (µg/L)
19.06.2017	267.8	7.70	39.3	2.8
20.06.2017		6.10	38.3	
21.06.2017	169.7	6.40	38	1
22.06.2017	164.9	5.40		
23.06.2017	118.2	4.50	37.3	
24.06.2017	107.2	5.30	36.4	0.3
25.06.2017	78.1	7.20	37	
26.06.2017	64.1	10.30	37.3	0.1
27.06.2017	63.7	11.40	36.5	
28.06.2017	56.2	10.50	36.6	
29.06.2017	42.3	21.60	36.5	
30.06.2017	62.4	15.30	36.7	0.1
01.07.2017		11.60	36.5	0.1
02.07.2017	81.1	11.00	36.6	
03.07.2017	54.6	10.50	36.1	
04.07.2017		9.40	36.4	
05.07.2017	43.1	8.50	36.5	
06.07.2017			36	
07.07.2017	39.9	8.70	37.1	
08.07.2017			37.1	
09.07.2017			36.1	
10.07.2017	32.9	7.4	36.5	
11.07.2017			36.3	
12.07.2017	29	7.8	36.6	
13.07.2017			36.6	
14.07.2017	37.3	6.8	36.5	
15.07.2017			36.5	
16.07.2017			36.8	
17.07.2017	24	7.4	36.2	
18.07.2017			36.7	
19.07.2017	24.4	7.7	36.7	
20.07.2017			36.2	
21.07.2017	38.6	9.6	37	
22.07.2017			37.5	
23.07.2017	46.5	9.3	36.6	
24.07.2017	43.4	8.9	36.1	
25.07.2017			36.7	
26.07.2017			37	
27.07.2017	18.2	8.6	37	
28.07.2017	15.1	8	37.1	

Date	sCRP (mg/L)	Leukocytes ($\times 10^9$/L)	Body temperature (°C)	sPCT (µg/L)
29.07.2017			37	
14.08.2017	16.1	8.3		
22.08.2017	9.9	6.5		

Red color shows days of phage therapy

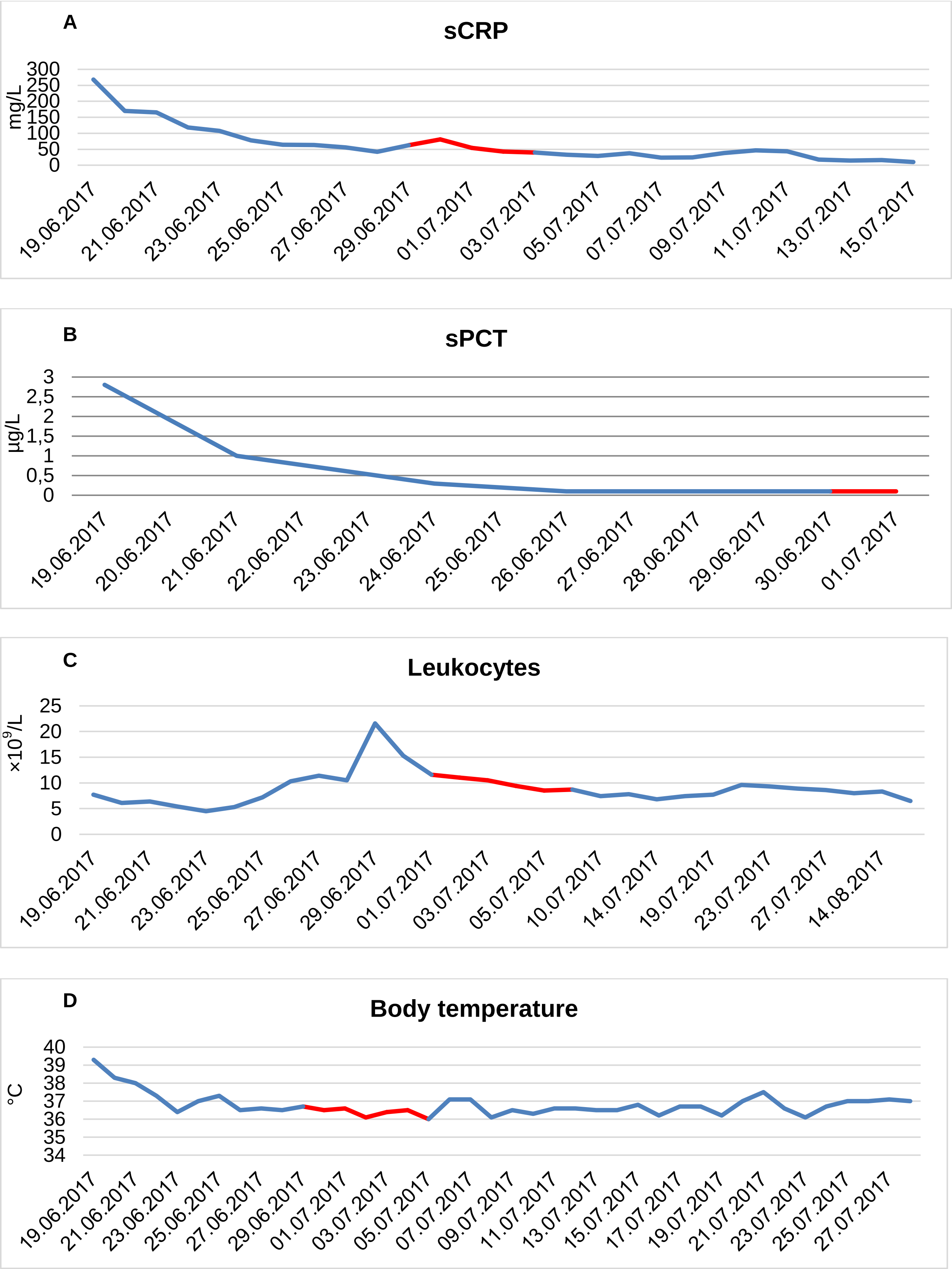

Supplementary Figure S4 – Patient 4

Supplementary Table S5 - Patient 5

Date	sCRP (mg/L)	Leukocytes (×10⁹/L)	Body temperature (°C)	sPCT (µg/L)
01.08.2017	47.3	10.5	37.8	
02.08.2017	56.1	14.9	37.3	
03.08.2017	84.9	16.6	36.6	1.4
04.08.2017	78.4	11.3	36.4	1.2
05.08.2017	53.6	8.7	35.8	
06.08.2017	42.7	8.1	36.6	
07.08.2017	32.8	6.8	35.8	
08.08.2017	29.8	8.3	36.5	
09.08.2017	23.2	8.3	35.7	
10.08.2017	19	8.2	35.5	0.2
11.08.2017	18.1	6.8	36.1	0.2
12.08.2017	18.6	5.4	36	
13.08.2017	20.8	6.6	35.8	0.3
14.08.2017	22.4	6.7	35.8	
15.08.2017	23	7.7	36.8	
16.08.2017	27	7.5	35	0.3
17.08.2017	33.2	7.6	36.6	0.4
18.08.2017	40	7.4	36	
19.08.2017	40.5	7	36.6	0.5
20.08.2017	37.2	6.5	36.2	0.6
21.08.2017	38.3	6.1	36.7	1
22.08.2017	40.9	7.7		
23.08.2017	36.2	6.5	35.7	
24.08.2017	35.3	7.8	36.7	
25.08.2017	36.5	8.3	36.9	
26.08.2017	52.5	9.2	36.4	
27.08.2017	61.4	9.1	36.5	
28.08.2017	56.3	9.4	36.5	
29.08.2017	46.5	9.1	36.8	
30.08.2017			35.9	
31.08.2017	48.2	10.2	36.1	

Red color shows days of phage therapy

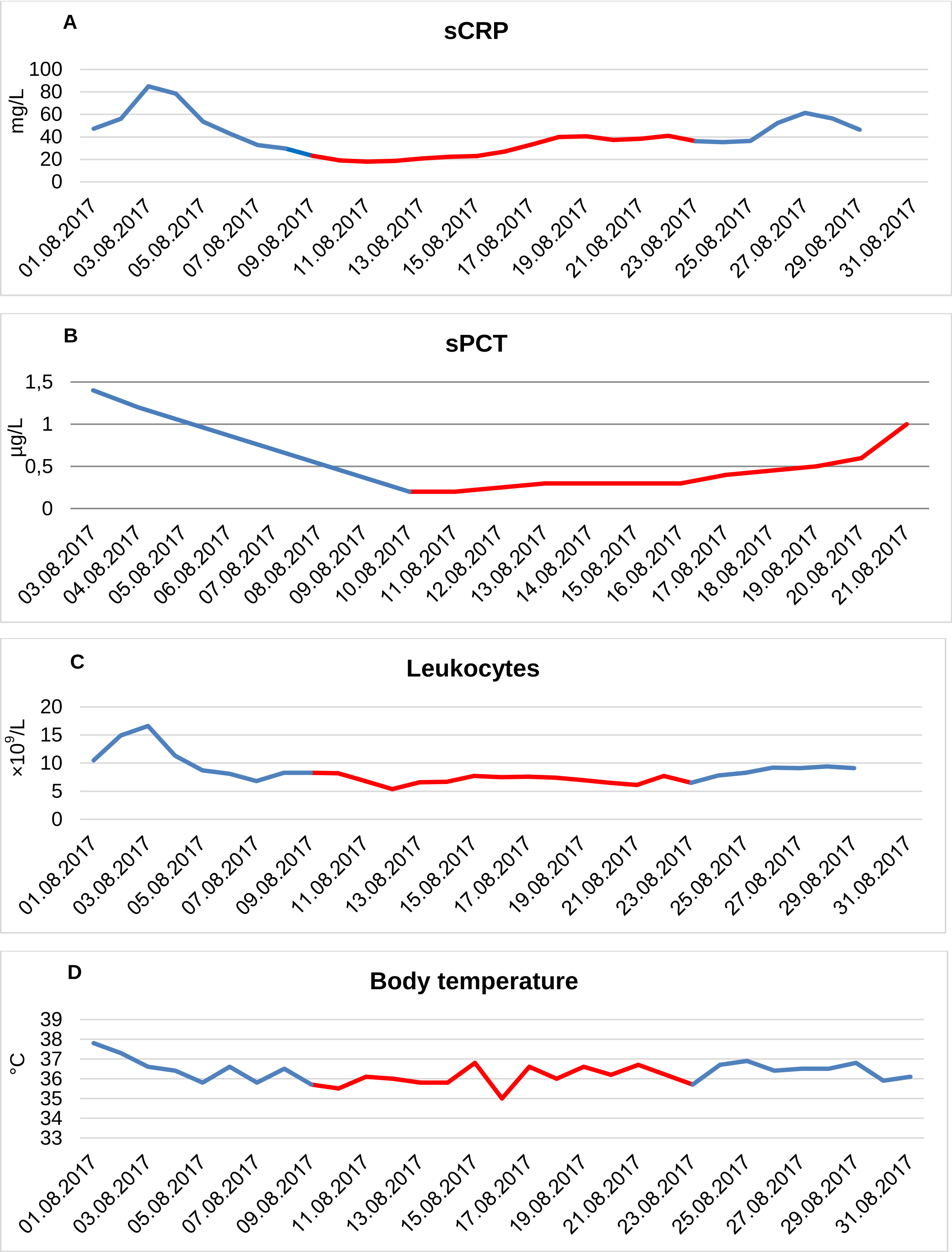

Supplementary Figure S5 – Patient 5

Supplementary Table S6 - Patient 6

Date	sCRP (mg/L)	Leukocytes ($\times 10^9$/L)	Body temperature (°C)	sPCT (µg/L)
11.11.2017	4	6.7		
12.11.2017				
13.11.2017				
14.11.2017				
15.11.2017				
16.11.2017	78.9	11.3	39.4	0.3
17.11.2017	189.9	11	38.3	
18.11.2017			36.4	
19.11.2017			35.6	
20.11.2017	37.6	6.9	36.3	
21.11.2017			36.5	
22.11.2017			36.4	
23.11.2017	10.4	6.2	36.2	
24.11.2017			35.4	
25.11.2017			36	
26.11.2017	6.8	5.5		<0.1
27.11.2017	6	6.1		<0.1
28.11.2017			36.1	
29.11.2017			36.5	
30.11.2017	13.1	8.9	37.4	
01.12.2017			36.6	
02.12.2017	37.4	7.2	36.4	
03.12.2017			35.9	
04.12.2017	15.2	7.8	36.6	
05.12.2017			36.6	
06.12.2017				
07.12.2017				
08.12.2017				
09.12.2017				
10.12.2017				
11.12.2017				
12.12.2017				
13.12.2017				
14.12.2017				
15.12.2017				
16.12.2017				
17.12.2017				
18.12.2017	3.2	8.1		

Red color shows days of phage therapy

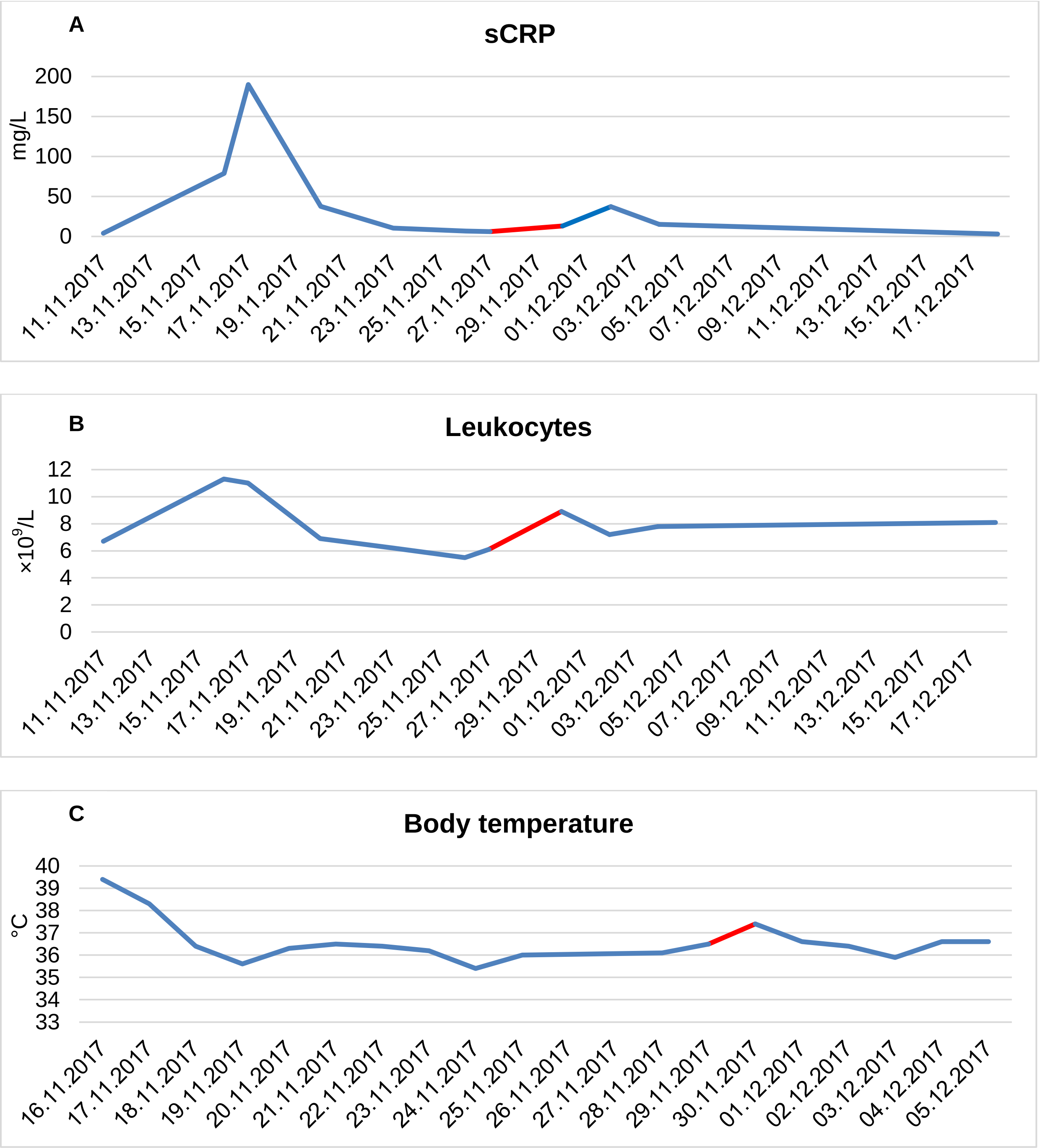

Supplementary Figure S6 – Patient 6

Supplementary Table S7 - Patient 7

Date	sCRP (mg/L)	Leukocytes (×10⁹/L)	Body temperature (°C)	sPCT (µg/L)
20.04.2018	304.8	9.8		
21.04.2018	265.8	6.5	37	
22.04.2018	208.4	5.7	36.8	
23.04.2018				
24.04.2018	120.1	7.7	36.6	
25.04.2018			36.5	
26.04.2018			36.6	
27.04.2018	86.2	11.1		
28.04.2018		10.3	37.1	
29.04.2018			36.6	
30.04.2018	79.4	12.3		
01.05.2018			36.2	
02.05.2018		9.1	36.3	
03.05.2018	62.2	8.9		
04.05.2018			36.3	
05.05.2018	74.8	8	36.6	
06.05.2018	45.1	6.7		
07.05.2018	40.9	6.9	36.2	
08.05.2018			36.1	
09.05.2018	31.9	7.9		
10.05.2018	35.2	10		
11.05.2018	176.3	9	37	
12.05.2018		8.8	36.4	
13.05.2018		8.6	36	
14.05.2018	82.8	10.7	36	
15.05.2018	59.6	8.7		
16.05.2018	34.5	7.1	36.3	
17.05.2018	28	7.4		
18.05.2018	29.6	8	35.9	
19.05.2018				
20.05.2018			36.3	
21.05.2018	22.8	9.2	36.1	
22.05.2018		10	36.2	
23.05.2018	21.5	9.5	36.1	
24.05.2018	24.1	9.6	35.8	
25.05.2018	24.5	9.2	36.5	

Red color shows days of phage therapy

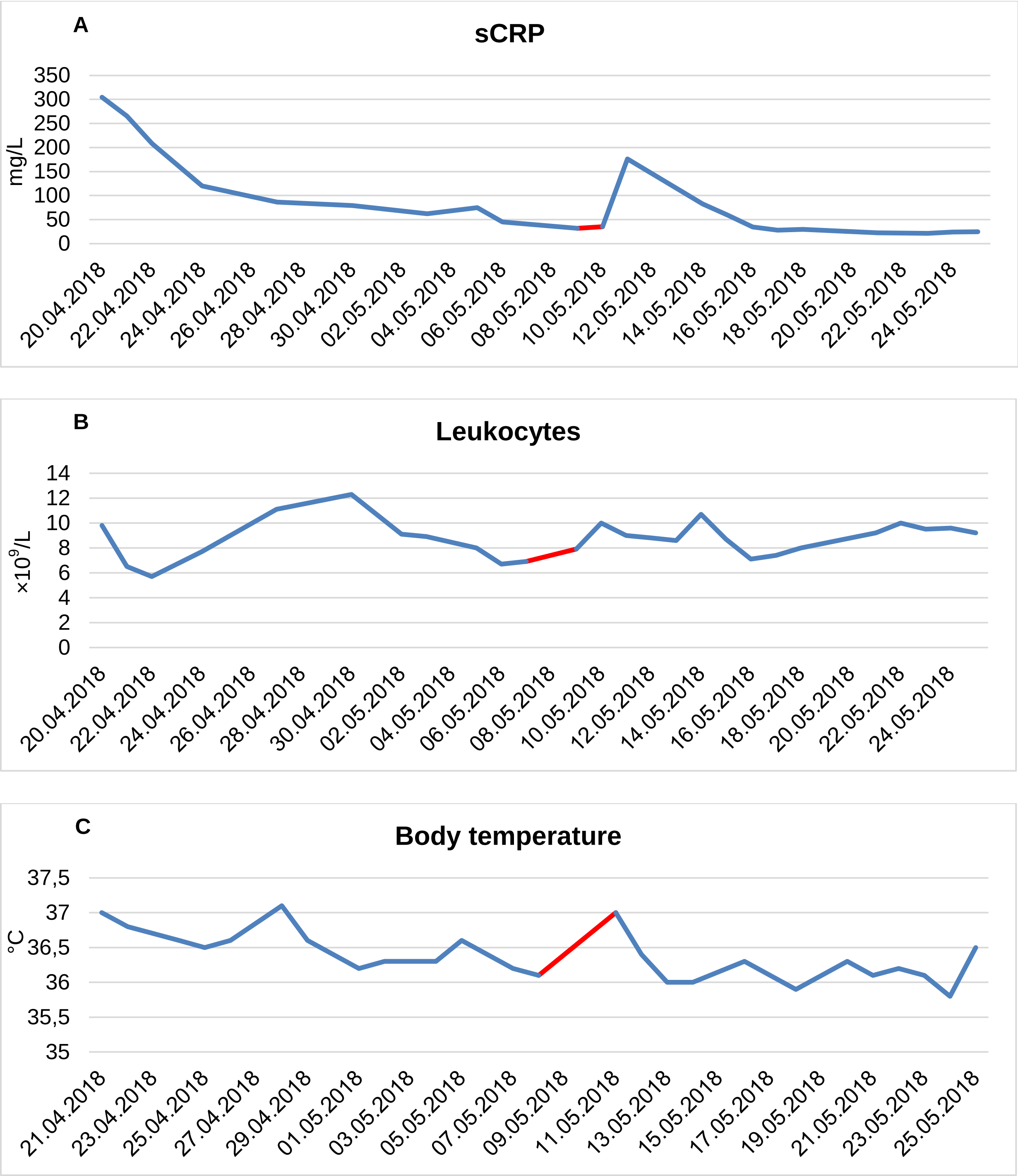

Supplementary Figure S7 – Patient 7

Supplementary Table S8 - Patient 8

Date	sCRP (mg/L)	Leukocytes ($\times10^9$/L)	Body temperature (°C)	sPCT (µg/L)
24.05.2018	50.7	5.8	37.3	
25.05.2018	117.2	5.8	37	<0.1
26.05.2018	113.4	4.8	37.2	<0.1
27.05.2018			37.5	
28.05.2018		4.2	36.3	
29.05.2018			36.6	
30.05.2018	55.7	3.2	36.2	
31.05.2018	20.3	2.8	36.6	
01.06.2018	11.6	3.3	36.3	
02.06.2018			36.6	
03.06.2018	4.3	3.5	36.3	
04.06.2018			36.5	
05.06.2018	1.7	3.5	36.5	
06.06.2018			36.2	
07.06.2018			36.8	
08.06.2018	0.4	3.4	36.4	
09.06.2018			36.2	
10.06.2018	0.4	3.5	36.4	
11.06.2018			36.4	
12.06.2018			35.8	
13.06.2018			35.7	
14.06.2018	0.7	3.5	36.7	
15.06.2018	1.1	3.9	36.2	
28.06.2018			36.4	
29.06.2018	0.4	4.2	36.5	

Red color shows days of phage therapy

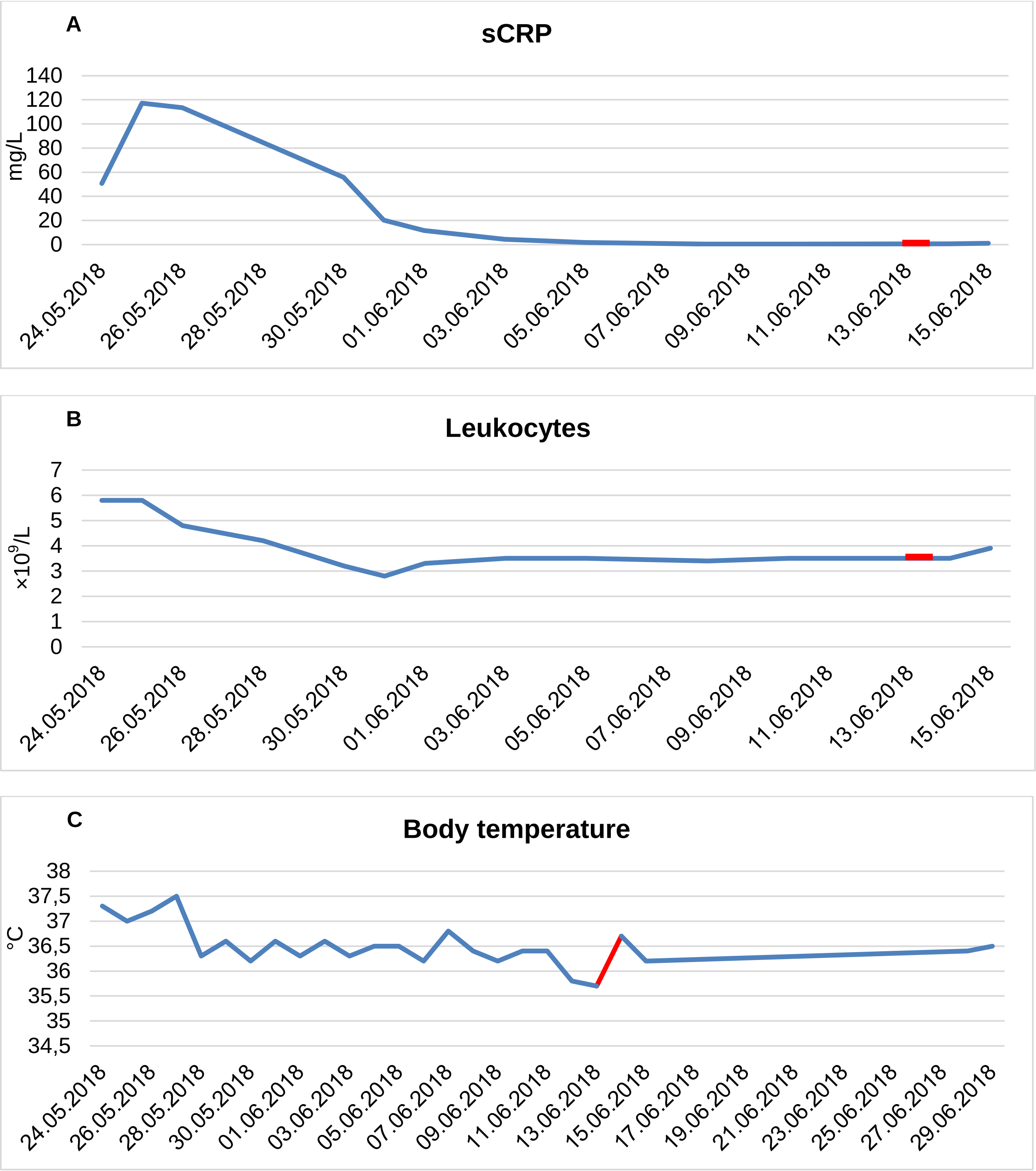

Supplementary Figure S8 – Patient 8

A

	[1]	
Ampicillin	R	>=32.0
Ampicillin-Sulbactam	R	>=32 0
Piperacillin / Sulbactam	R	
Piperacillin-Tazobactam	R	>=128.0
Cefuroxim	R	>=64 0
Cefuroxim-Axetil	R	>=64 0
Ceftriaxon	R	
Cefotaxim	R	>=64 0
Ceftazidim	R	>=64 0
Gentamicin	R	>=16.0
Doxycycline	R	
Tetracyclin	R	>=16.0
Levofloxacin	R	
Ciprofloxacin	R	>=4 0
Moxifloxacin	R	>=8.0
Meropenem	R	>=16.0
Ertapenem	R	>=8.0
Cotrimoxazol	R	>=320.0

B

	[1]		[2]	
Ampicillin	R	>=32.0	R	>=32.0
Ampicillin-Sulbactam	R	>=32.0	R	>=32.0
Piperacillin / Sulbactam	R		R	
Piperacillin-Tazobactam	R	>=128.0	R	
Cefuroxim	R	>=64.0	R	16.0
Cefuroxim-Axetil	R	>=64.0	R	16.0
Cefpodoxim	R	>=8.0	R	2.0
Ceftriaxon	R		R	
Cefotaxim	R	>=64.0	R	<=1.0
Ceftazidim	R	>=64.0	R	<=1.0
Gentamicin	R	>=16.0	R	>=16.0
Tigecyclin	R	4.0	R	4.0
Doxycycline	R		R	
Tetracyclin	R	>=16.0	R	>=16.0
Levofloxacin	R		R	
Ciprofloxacin	R	>=4.0	R	>=4.0
Moxifloxacin	R	>=8.0	R	>=8.0
Meropenem	S	<=0.25	S	<=0.25
Ertapenem	S	<=0.5	S	<=0.5
Cotrimoxazol	R	>=320.0	R	>=320.0

Supplementary Figure S9 – Antibiogram from Patient 2: (A) *K. pneumoniae* isolated from lungs prior phage therapy; (B) *K. pneumoniae* isolated from stool after phage therapy
